# Long-Term Consequence of Non-neurotropic H3N2 Influenza A Virus Infection for the Progression of Alzheimer’s Disease Symptoms

**DOI:** 10.3389/fncel.2021.643650

**Published:** 2021-04-28

**Authors:** Shirin Hosseini, Kristin Michaelsen-Preusse, Klaus Schughart, Martin Korte

**Affiliations:** ^1^Department of Cellular Neurobiology, Zoological Institute, TU-Braunschweig, Braunschweig, Germany; ^2^Neuroinflammation and Neurodegeneration Group, Helmholtz Centre for Infection Research, Braunschweig, Germany; ^3^Department of Infection Genetics, Helmholtz Centre for Infection Research, Braunschweig, Germany; ^4^Department of Infection Genetics, University of Veterinary Medicine Hannover, Hanover, Germany; ^5^Department of Microbiology, Immunology and Biochemistry, University of Tennessee Health Science Center, Memphis, TN, United States

**Keywords:** Alzheimer’s disease, influenza virus, hippocampus, microglia, synaptic plasticity, behavior

## Abstract

Influenza viruses until today are a leading cause of worldwide severe pandemics and represent a major threat to human and animal health. Although the primary target of influenza viruses is the lung, infection may manifest with acute and even chronic neurological complications (e.g., status epilepticus, encephalopathies, and encephalitis) potentially increasing the long-term risk for neurodegenerative diseases. We previously described that a peripheral influenza A virus (IAV) infection caused by non-neurotropic H3N2 (maHK68) variant leads to long-term neuroinflammation and synapse loss together with impaired memory formation in young adult mice. Processes of neuroinflammation have been associated with neurodegenerative diseases such as Alzheimer’s disease (AD) and prolonged or excessive innate immune responses are considered a risk factor for AD. Here, the role of purely peripheral IAV infection for the development and progression of AD in a transgenic mouse model (APP/PS1) was investigated. At 2 months of age, mice were infected with H3N2 IAV and the detailed analysis of microglia morphology revealed neuroinflammation in the hippocampus already of 6 months old non-infected APP/PS1 mice together with impaired spatial learning, however, microglia activation, amyloid-β plaques load and cognitive impairments were even more pronounced in APP/PS1 mice upon H3N2 infection. Moreover, CA1 hippocampal dendritic spine density was reduced even at 120 dpi compared to wild-type and also to non-infected APP/PS1 mice, whereas neuronal cells number was not altered. These findings demonstrate that non-neurotropic H3N2 IAV infection as a peripheral immune stimulation may exacerbate AD symptoms possibly by triggering microglial hyperactivation.

## Introduction

Alzheimer’s disease (AD) is one of the most common forms of dementia characterized by amyloid-β plaques, neurofibrillary tangles, chronic neuroinflammation, gliosis and eventually neuronal cell death (DeTure and Dickson, 2019). Although AD is very prevalent especially in people above the age of 65, efficient therapeutic interventions are lacking because of the complex nature of the disease. Importantly, until now the detailed cellular mechanisms responsible for disease development and progression especially with respect to genetic vs. environmental risk factors have yet to be fully understood (Liu et al., 2019). Among the environmental risk factors increasing evidence points toward infection and subsequent neuroinflammation as a trigger for AD. For example, Bowery et al. showed that intra-hippocampal tetanus toxin administration leads to neurodegeneration in rats (Bowery et al., 1992). In fact, the tetanus toxin via excitotoxicity and subsequent inflammatory reactions causing neurodegeneration (Jiruska et al., 2013; Rana and Musto, 2018). In addition, there is evidence of direct viral infections caused by persistent neurotropic viruses of the central nervous system (CNS) such as human immunodeficiency virus (HIV-1), human herpesviruses (HHV), Japanese encephalitis virus (JEV) and herpes simplex virus type-1 (HSV-1) being involved in the etiology of neurodegenerative diseases (Perkins, 2005). Viral particles were indeed detected in brain autopsies or cerebrospinal fluid from patients diagnosed with neurodegenerative diseases (De Chiara et al., 2012). Such persistent CNS infections usually have a relatively long incubation period and a chronic progression, therefore, given the increasing life-span their involvement in triggering or enhancing neurodegeneration might increase.

The direct interaction between viral infections and particularly AD has long been a subject of interest, however, so far direct causative effects could not be proven. In addition to persistent viral infections as a potential risk factor (Allnutt et al., 2020), acute respiratory viruses such as influenza A viruses (IAV) and coronaviruses (CoV) have repeatedly been suggested to be involved in the etiology of neurodegenerative diseases (Bohmwald et al., 2018). Yet, any causality between acute respiratory viral infection and neurodegenerative diseases is challenging to establish, in part due to the potential lag time between acute infection and the subsequent development of pathology. In line with this, after the Spanish flu (1918–1920), patients developed symptoms of neurodegeneration such as Parkinsonism even decades later (Ekstrand, 2012).

IAVs are one of the most critical challenges for public health, as every year they are responsible for high rates of morbidity and mortality, mainly among young children, elderly and patients with immunodeficiency. Besides the primary infection of the respiratory tract, neurological complications such as encephalitis, meningitis and seizures have been also reported in IAV infected individuals (Ekstrand, 2012). Some IAV strains (i.e., H7N7 and H5N1) are neurotropic, and therefore can directly invade the CNS through different routes, such as infecting microvascular endothelial cells, ascending the olfactory, trigeminal or vagus nerves where they can infect neurons and other resident cells of the CNS thereby leading to neurodegeneration and neural dysfunction (Bohmwald et al., 2018). However, also strains that remain purely in the periphery (non-neurotropic) can induce neurological complications as for instance the strain of H1N1 influenza involved in the 2009 swine flu, for which a well-documented association with encephalitis was described (Sadasivan et al., 2015).

In line with this, we could recently show that influenza infection of 2 months old wild-type mice with neurotropic H7N7 as well as non-neurotropic H3N2 IAV subtypes can initiate inflammatory cascades via microglia activation in the hippocampus associated with synapse loss, impaired synaptic plasticity as well as deficits in spatial memory formation well beyond the acute phase of disease at 30 days post infection (dpi) (Hosseini et al., 2018). Indeed, these findings show that influenza infection with a neurotropic but also non-neurotropic viral strain may induce excessive and persistent microglial activation and subsequent neuroinflammation in the CNS, thus increasing the likelihood of developing neurodegenerative diseases.

In the current study, given the long-term neuroinflammation, as a central mechanism contributing to AD, induced by especially non-neurotropic IAV virus on hippocampal morphology and function (Hosseini et al., 2018), we aimed to investigate the role of H3N2 IAV infection for the development and progression of AD symptoms in an APP/PS1 transgenic mouse model. Our findings revealed that microglial reactivity was enhanced within the hippocampus of H3N2 IAV infected APP/PS1 mice. This increase was associated with greater amyloid-β plaques load, synapse loss, and cognitive decline in these mice at 120 dpi.

## Materials and Methods

### Animals

In this study, 2 months old female APPSwe/PSEN1dE9 transgenic mice which over express the Swedish mutation of APP together with PS1 deleted in exon 9 (Mutations: APP KM670/671NL (Swedish), PSEN1: deltaE9, MMRRC Stock No: 34832-JAX) and littermate control mice, also referred to as wild-type (WT), were used. Mice were bred and kept under specific-pathogen-free conditions, on a 12 h light/dark cycle with *ad libitum* access to water and food in the central mouse facility of the Helmholtz Centre for Infection Research, Braunschweig. All experimental procedures have been assessed and approved by the local committees at the Helmholtz Centre for Infection Research and TU Braunschweig and the authorities (LAVES, Oldenburg, Germany; permit number: 18/2968) according to the national guidelines of the animal welfare law in Germany.

### Virus Preparation and Infection

Stocks of maHK68, mouse-adapted A/Hong-Kong/1/68 (H3N2) influenza A virus were obtained from Georg Kochs, University of Freiburg (Haller et al., 1979) and propagated by infection of 10 days old embryonated chicken eggs (Charles River Laboratories) in the lab of Klaus Schughart, Helmholtz Centre for Infection Research. For infection procedure, 8–10 weeks old mice were anesthetized using intraperitoneal (i.p.) injection of ketamine-xylazine solution (85% NaCl (0.9%), 10% ketamine (100 mg/ml), 5% xylazine (20 mg/ml); 10 ml per 1 kg body weight) and then infected intranasally with a sublethal low dose of 10 FFU (focus forming units) of virus in 20 μl sterile phosphate-buffered saline (PBS 1X) (Hosseini et al., 2018; Kollmus et al., 2018). The bodyweight and physical appearance including (spontaneous behavior, posture, fur and behavior upon provocation) were assessed for 14 consecutive days after infection to confirm the successful infection and subsequent recovery of survived animals. The percentage of body weight loss was calculated in comparison with the bodyweight on day 0 (prior to infection). Mice with more than 30% body weight loss or severe physical complications were euthanized for ethical reasons. In control groups (non-infected) mice were intranasally inoculated with 20 μl sterile PBS 1X (Hosseini et al., 2018).

### Congo-Red Staining

Brains hemispheres of experimental naïve animals were isolated and fixed in 4% paraformaldehyde (PFA) for 24 h and then cryoprotected in 30% sucrose solution in PBS1X for additional 24 h and stored in Tissue-Tek optimum cutting temperature compound (Hartenstein Laborversand) at −70°C. Right hemispheres were used for Congo-red staining and the left hemispheres were kept for additional immunohistochemistry. The 20 μm sections were cut using Leica Cryostat (CM3050 S) and five successive sections after washing with PBS 1X were placed on gelatin-coated slides. For Congo-red staining, FD Congo Red Solution^TM^ kit (FD Neurotechnologies, Inc.) was used according to the manufacturer’s protocol and finally, the slices were mounted using Permount (Thermo Fisher Scientific).

### Immunohistochemistry

Left hemispheres after fixation and cryoprotection procedures were cut using Leica Cryostat in 20 μm thickness (see section “Congo-Red Staining”). Five successive sections from each hemisphere were washed with PBS1X. Sections were transferred into a blocking solution containing 0.2% Triton X-100, 10% goat serum and 1% bovine serum albumin (BSA) in PBS 1X for 1 h at room temperature. Then, sections were incubated overnight at 4°C with the primary antibodies including anti-ionized calcium-binding adaptor molecule 1 (IBA-1) (1:1,000; rabbit polyclonal; Synaptic Systems, Cat#234 003, RRID:AB_10641962) and anti-NeuN, clone A60 (1:500; mouse monoclonal; Merck Millipore, Cat#MAB377, RRID:B_2298772) diluted in PBS 1X, 0.2% Triton X-100, and 10% goat serum. After 1 h washing with PBS 1X, the sections were incubated with secondary antibodies diluted in PBS 1X including Cy^^TM^3-conjugated AffiPure goat anti-rabbit IgG (H + L) (1:500; Jackson ImmunoResearch Laboratories, Cat#111-165-144, RRID:AB_2338006) and Cy^TM^2-conjugated AffiPure goat anti-mouse IgG (H + L) (1:500; Jackson ImmunoResearch Laboratories, Cat#115-225-146, RRID:AB_2307343) for 2 h at room temperature. Afterward, sections were washed by PBS 1X thoroughly for 1 h and stained with 4’,6-diamidino-2-phenylindole (DAPI) (Sigma-Aldrich) subsequently followed by mounting with Fluoro-gel medium (Electron Microscopy Sciences, Hatfield, PA).

### Golgi-Cox Staining

For Golgi-Cox staining, FD Rapid GolgiStain^TM^ Kit (FD Neurotechnologies, Inc.) was used according to the manufacturer’s protocol. Brains hemispheres of experimental naïve animals were incubated in Golgi solutions mixture. Hemispheres were placed in 2% agar. Then coronal sections with 200 μm thickness were cut using Leica Vibratome (VT 1000 S) and mounted on gelatin-coated slides. The sections were further processed for signal development and finally, mounted using Permount (Thermo Fisher Scientific).

### Imaging and Image Analysis

Congo-red stain exhibits a bright fluorescence emission at 614 nm with excitation at 497 nm. The two-dimension frames of Congo-red stained brain sections were taken using an Axioplan 2 imaging microscope (Zeiss) with 2.5X objective (N.A. 0.07) connected to a digital camera (AxioCam MRm, Zeiss) with the same fluorescent light exposure time (700 ms) in all groups. In the whole hippocampus, the amyloid-β plaques area was quantified using Fiji software (BioVoxxel). Using the polygon selection tool, the area of hippocampus was measured and then the amyloid-β stained area was calculated using the analyze particle tool. Total Congo-red positive areas were quantified in the hippocampus and the final data was expressed as the ratio of amyloid-β-positive area/total hippocampal area multiple 100.

Images obtained from the anti-IBA-1 and anti-NeuN staining in the CA1 hippocampal sub-region were collected using an Axioplan 2 imaging microscope (Zeiss) equipped with an ApoTome module (Zeiss) in three-dimensions (z stack thickness of 1 μm) with 20X objective (N.A. 0.8) connected to a digital camera (AxioCam MRm, Zeiss). To quantify the density of microglia and mature neurons, a region of interest (ROI) was drawn in each frame of the hippocampal CA1 sub-region and the total number of IBA-1 and NeuN positive cells with clearly visible nuclei were counted manually by Fiji software (BioVoxxel). ROI volume was calculated and results were quantified as the number of cells per mm^3^ tissues and then normalized to control. For morphometric analysis of IBA-1 positive cells, at least 30 microglial cells (6 cells per each ROI) were randomly selected in CA1 hippocampal sub-region of anti-IBA-1 stained images from each animal. The total number of primary microglial cell processes as a morphological index for reactivity (Papageorgiou et al., 2015) was counted using Fiji software (BioVoxxel).

In Golgi-Cox stained slices, second- or third-order branches of apical and basal dendrites located in the hippocampal CA1 sub-region were imaged in three-dimensions (z stack thickness of 0.5 μm) using an Axioplan 2 imaging microscope (Zeiss) equipped with a 63X (N.A. 1.4) oil objective connected to a digital camera (AxioCam MRm, Zeiss). In the randomly selected neurons, spine density per micrometer dendrite with a length of more than 60–70 μm which were located at least 50 μm away from the soma, was calculated using Fiji software (BioVoxxel).

### Electrophysiological Experiments

Brains were rapidly isolated and kept in the ice-cold carbogenated (95% O_2_ and 5% CO_2_) artificial cerebrospinal fluid (ACSF) containing (in mM): 124 NaCl, 4.9 KCl, 1.2 KH_2_PO_4_, 2.0 MgSO_4_, 2.0 CaCl_2_, 24.6 NaHCO_3_, and 10 D-glucose, pH 7.4 (one hemisphere was used for Golgi-Cox staining). Then 400 μm hippocampal acute sections were cut using a manual tissue chopper. The acute hippocampal slices were placed in an interface recording chamber (Scientific System Design), where they were kept at 32°C with a constant flow rate (0.5 ml/min) of carbogenated ACSF for 2 h prior to the recording.

Field excitatory postsynaptic potentials (fEPSPs) were measured in the stratum radiatum of the CA1 hippocampal sub-region in acute slices. Responses were evoked by electrical stimulation of the Schaffer collateral pathway connecting CA3 to CA1 using a monopolar, lacquer-coated stainless-steel electrodes (5 MΩ; AM Systems). For recording fEPSPs (recorded as the first slope function), the recording electrode (5 MΩ; AM Systems) was placed in the CA1 apical dendritic layer at least 20 μm far from the stratum pyramidale and signals were amplified by a differential amplifier (Model 1700, AM Systems). The signals were digitized using a CED 1401 analog-to-digital converter (Cambridge Electronic Design). An input-output curve (afferent stimulation vs. fEPSP slope) for measurement of basal synaptic transmission was assessed 2 h after pre-incubation. Stimulus intensity was adjusted to obtain fEPSP slope as 40% of the maximal fEPSP response. To study short-term plasticity, a paired-pulse stimulation protocol was done using two consecutive stimuli with equal intensity from the stimulating electrode in each hippocampal slice. Paired-pulse facilitation (PPF) was extracted from the fEPSP slopes as a response to the second stimulus over the first one at different interpulse intervals of 10, 20, 40, 60, 80, and 100 ms. To investigate long-term potentiation (LTP), 20 min after stable baseline recording LTP was induced by Theta-burst stimulation (TBS) including four bursts at 100 Hz repeated 10 times in a 200 ms interval. This stimulation was repeated three times in a 10 s interval. The slope of fEPSPs was measured over 60 min and normalized to the baseline. Data acquisition and offline analysis were performed using IntraCell software (Version 1.5, LIN, Magdeburg, 2000) (Hosseini et al., 2018).

### Behavioral Experiments

The behavioral experiments were operated during the light period between 9:00 and 16:00 under dim light illumination. The repeated tests were performed almost at the same time of day.

#### Open Field Test

Spontaneous locomotor activity and willingness to explore of the mice were assessed using the open field test as previously described (Walsh and Cummins, 1976). Mice were placed along one side of a white PVC open field apparatus (40 cm × 40 cm × 40 cm) for 5 min. The central area of the arena was specified as the center part (30 cm × 30 cm). Between each session of experiments, the apparatus was completely cleaned with Bacillol^®^ to reduce the odor cues. Movement data including total distance traveled, average speed and percentage of mice activity in the periphery and center parts of the arena were collected by ANY-maze (Stoelting, Dublin, Ireland) behavioral tracking software.

#### Morris Water Maze Test

To assess the cognitive function, spatial learning and memory formation was studied using initial and reversal learning in Morris water maze test. Spatial learning in this test is considered as a hippocampus-dependent task where the animals should navigate a hidden platform with the help of visual cues surrounding the water basin (Morris, 1984). The maze was comprised of a circular basin (150 cm diameter) filled with 19–20°C water and colored opaque with non-toxic white paint (Titaandioxide, Euro OTC Pharma).

The transparent platform (10 cm diameter) was hidden 1 cm underneath the surface of the opaque water in the target quadrant of the maze with around 35 cm distance to the basin wall. Visual cues including circle, triangle and 4 vertical stripes were adjusted an average distance of 30 cm to the maze. Prior to the training, 3 days of training (two trials, 60 s each) with a visible platform task were performed as pre-training task which was utilized to ensure that swimming ability and visual acuity were intact in all animals. In addition, pre-training test was important for the animals to get used to the test situation without any significant stress. Here the animals did not show any significant difference in swimming speed and arriving time to a visible platform (data are not shown).

Subsequently, initial learning was done for 8 consecutive days with the hidden platform located in the northeast (NE) quadrant. Each training day included four trials (60 s each) with different starting points (SE, S, W, and NW) randomly with at least 5 min intertrial interval. The animals were allowed to swim freely for 60 s or until the platform was reached. Otherwise, they were guided to the platform and permitted to sit on it for 20 s. The videos were recorded with a camera in the ceiling at the top of the basin. The swim speed of mice was comparable between all groups (data are not shown).

To assess the qualitative aspects of spatial acquisition during the 8 days of training, the swimming pathway to navigate the hidden platform was analyzed as the searching strategy. Over time, mice switch from egocentric hippocampus-independent strategies including random searching characterized by > 80% coverage of the surface, scanning characterized by > 75% presence in the center part of the basin and chaining characterized by > 75% swimming in the doughnut-shaped annulus zone to an allocentric direct swimming strategy characterized by > 50% presence in the Wishaw’s corridors (goal corridor) which depends on the hippocampus (Figure 5F; Hosseini et al., 2020).

To study the reference memory, three probe trial tests were done on day 3 and 6 prior to the acquisition training and on day 9, 24 h after the last day of training session. During the probe trials, the platform was removed and the animals were permitted to swim freely for 45 s.

Twenty four hours after the last probe trial, the reversal learning was performed. For this purpose, the platform was moved to the opposite quadrant of the basin (SW) to investigate the ability of the mice to form a new memory which competes with the previous memory of the old platform position. The reversal learning required plasticity and flexibility of behavior (Vorhees and Williams, 2006). Afterward, on day 4, the single probe trial was done.

All data including the escape latency (time to reach the platform), percentage of searching time spent in the different quadrants of the pool, percentage time spent in the border (thigmotaxis), annulus (chaining), central circle (scanning) zones and Whishaw’s corridors (direct swimming) of the basin, and occupancy plot of the presence in the quadrants for the animals groups were collected by ANY-maze (Stoelting, Dublin, Ireland) behavioral tracking software.

### Statistical Analysis

Data were presented as mean ± SEM and analyzed and plotted by GraphPad Prism 9 (GraphPad Software, Inc., United States). Differences of microglia density and activity, amyloid-β plaques load, neuronal and dendritic spine density data were subjected to a two-way ANOVA (Two variable factors: Genotype and infection) or a Student’s *t*-test whereas repeated measure two-way ANOVA was used for behavioral and electrophysiological experiments. Fisher’s LSD multiple comparison was used as a *post hoc* test. The minimum significance value was considered as *p* < 0.05. The minimum animal numbers in all experiments were calculated *a priori* using G^∗^Power 3.1.9.4 software (Heinrich Heine University Düsseldorf, Germany). The “N” in different experimental groups were reported in the respective figure legends. All experiments were analyzed in a strictly blind fashion.

## Results

### Influenza A Virus Infection in APP/PS1 Mice–Long-Term Consequences of Acute Viral Infection for the Progression of AD Symptoms

**FIGURE 1:**
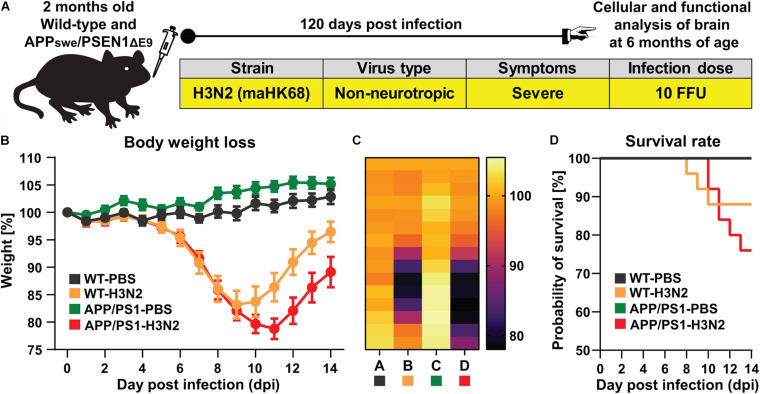
H3N2 IAV infection in APPswe/PSEN1dE9 mouse model. **(A)** 2 months old wild-type (WT) and APP/PS1 mice were infected intranasally with 10 FFU non-neurotropic H3N2 IAV subtype. **(B)** A representative experiment of body weight loss depicted as a percentage of the starting weight of mice during the acute phase of IAV infection is shown (*N* = 15 in each group); The body weight loss curve as well as **(C)** the heat map of body weight loss during 14 days in each group clearly indicated that APP/PS1 mice lost more weight and recover slower compared to WT infected mice. **(D)** The survival rate of control and infected mice is presented. 100% of control (non-infected WT and APP/PS1 mice) survived while only 88% of WT mice and 76% of APP/PS1 survived from H3N2 IAV infection (*N* = 25 in each group).

In this study, 8–10 weeks old APPswe/PS1ΔE9 and wild-type (WT) mice were intranasally infected with the well-characterized non-neurotropic mouse-adapted human influenza A virus (IAV) strain maHK68 (H3N2) (Haller et al., 1979; Leist et al., 2016; Kollmus et al., 2018). Previously, we demonstrated that infection with this non-neurotropic subtype leads to impairments in hippocampal function and structure well beyond the acute phase of the disease still detectable at 30 and even partially at 60 days post infection (dpi) in C57BL/6J WT mice (Hosseini et al., 2018). Full recovery could only be observed at 120 dpi in WT animals. Therefore, we were interested in whether infection with this variant which notably does not affect neurons directly may indeed result in more severe and prolonged impairments in the AD mouse model. Comparable to our previous study, a sub-lethal low dose (10 FFU) of the virus was used which allowed us to investigate long-term effects of H3N2 IAV infection (after 120 days) (Figure 1A). Control groups were inoculated intranasally with an equal amount of sterile PBS 1X as virus vehicle. The bodyweight and general behavior of all mice were monitored for 14 days post infection to confirm successful infection and subsequent recovery of all surviving animals. Indeed, bodyweight loss of rodents can be considered as an index for pathogen burden and cytokine release following many infectious conditions (van Heeckeren et al., 2000). After 3–4 days, both WT and APP/PS1 IAV infected mice started to lose bodyweight and exhibited disease behavior with overall reduced activity, ruffled fur and diminished food and water intake (Figures 1B,C).

While the maximum percentage of body weight loss was observed at 9 dpi in WT mice (83 ± 3% of the initial bodyweight), this was more pronounced and prolonged in H3N2 IAV infected APP/PS1 animals (at 11 dpi, 79 ± 2% of the initial bodyweight, Figures 1B,C). While all surviving infected mice recovered, this process was delayed in IAV infected APP/PS1 mice. Moreover, following H3N2 IAV infection, the survival rate of APP/PS1 mice (∼76%) was lower than WT mice (∼88%) (Figure 1D). Overall these results suggest that even young adult APP/PS1 mice are more susceptible to respiratory viral infection, as the weight loss was greater and recovery slower than in WT mice.

### H3N2 IAV Infection Chronically Increases Microglia Reactivity and Amyloid-β Plaque Burden in the Hippocampus of APP/PS1 Mice

Microglia are the major resident immune cell type in the CNS which not only react to the direct presence of a pathogen in the brain but also to peripheral immune stimuli (Jurgens et al., 2012; Vasek et al., 2016; Hosseini et al., 2018). Activation of microglia and subsequent acute inflammation in the brain is a well-established defense line against infection. However, if inflammatory signaling is not terminated but rather persists for a prolonged period of time, chronic neuroinflammation can occur, characterized by a chronic increase in microglia density and activity (Wolf et al., 2017). Sustained proliferation and activation of microglia, along with other infiltrating immune cells, has been shown to exacerbate both amyloid-β and tau pathology and may serve as an important contributor to the pathogenesis of AD (Kinney et al., 2018).

**FIGURE 2:**
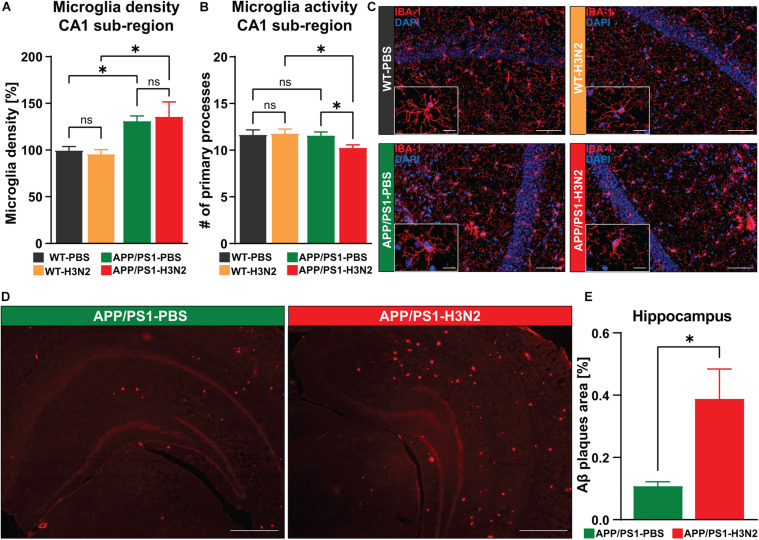
H3N2 IAV infection increases the microglial activation status and amyloid-β plaque burden within the hippocampus of APP/PS1 mice. **(A)** At 120 days post H3N2 IAV infection the microglia density in the CA1 hippocampal sub-region was comparable between infected and non-infected WT mice. Microglia density was increased significantly in non-infected APP/PS1 mice compared to WT, however, H3N2 IAV infection in APP/PS1 mice did not lead to a further elevation in microglial cell populations [N (number of animals in each group) = 3]. **(B)** H3N2 IAV infection led to a reduced number of microglial primary processes within the hippocampal CA1 sub-region of APP/PS1 mice but the other tested groups did not show any significant differences (*N* = 3, ∼30 randomly selected microglia were analyzed per animal). **(C)** Representative examples of IBA-1 immunostaining at 120 dpi (20X); Scale bar = 50 μm. Inserts, higher magnifications of the respective images; Scale bar = 10 μm. **(D)** Representative examples of Congo-red staining at 120 dpi (2.5X); Scale bar = 400 μm. **(E)** At 120 dpi, the area occupied with amyloid-β was increased in the hippocampus of H3N2 IAV infected APP/PS1 mice compared to non-infected (*N* = 3). Data are presented as mean ± SEM, for each individual 5 sections were taken and data are presented from averaging the values of the sections per animal. *p < 0.05.

Therefore, to identify the potential role of H3N2 IAV infection in the onset and progression of AD-related pathologies in APP/PS1 mice, microglial density and reactivity were examined in the hippocampus of 6-month-old animals at 120 dpi. AD-related pathologies usually begin in APPswe/PS1ΔE9 mice at approximately 6–7 months of age (Trinchese et al., 2004). For this purpose, anti-IBA-1 staining was performed (Figures 2A–C). First, IBA-1 positive cells were quantified in the CA1 sub-region of the hippocampus which is the first hippocampal area described to be affected in AD (Masurkar, 2018).

Consistent with our previous findings, microglia density was not chronically altered in infected WT mice compared to non-infected WT mice (Δ 4%, *p* = 0.75) (Hosseini et al., 2018). Moreover, as in WT mice, infection with H3N2 IAV did not further significantly increase microglial density in the hippocampus of infected APP/PS1 mice compared to non-infected APP/PS1 mice (Δ 5%, *p* = 0.71), [Two-way ANOVA *F*_Infection (1, 8)_ = 0.0018, *p* = 0.96]. Interestingly, IBA-1 positive cells were already significantly increased in the hippocampus of non-infected APP/PS1 mice compared to WT animals (Δ 31%, *p* = 0.03). This was also the case for H3N2 IAV infected APP/PS1 compared to infected WT mice (Δ 39%, *p* = 0.013), [Two-way ANOVA *F*_Genotype (1, 8)_ = 15.93, *p* = 0.004, Figure 2A].

In the healthy CNS, microglial cells exhibit a sophisticated cell architecture with numerous fine processes constantly scanning the microenvironment. Under pathological conditions, however, these “resting” microglia change their shape into an active form by retracting processes thereby displaying a more and more round cell shape (Papageorgiou et al., 2015). An assessment of the number of primary processes can therefore be used to determine the microglial activation status under healthy conditions or following infection with H3N2 IAV. In WT animals no chronic reduction in the number of primary processes was observed at 120 days post infection with H3N2 (Δ 1%, *p* = 0.81). Also, PBS-treated APP/PS1 mice did not show significant alterations in the activation status compared to WT mice (Δ 1%, *p* = 0.80) (Figure 2B). However, 120 days post H3N2 IAV infection in APP/PS1 mice the number of primary processes was significantly reduced compared to infected WT (Δ 12%, *p* = 0.02) and non-infected APP/PS1 animals (Δ 10%, *p* = 0.04), [Two-way ANOVA *F*_Genotype (1, 8)_ = 5.08, *p* = 0.05, Figures 2B,C].

It was shown that the interaction of microglia with amyloid-beta plaques enhances the microglial reactivity (Mandrekar-Colucci and Landreth, 2010), therefore to identify the association between increased microglial activation in the H3N2 IAV infected APP/PS1 mice and amyloid-β accumulation during the progression of AD, the amyloid-β plaque load was examined using Congo-red staining (Figures 2D,E). The results showed that amyloid-β plaques occupied a larger area in the hippocampus of infected APP/PS1 mice at 120 dpi compared to non-infected ones (Unpaired *t*-test: *t* = 2.90, df = 4, *p* = 0.044, Figure 2E).

Taken together, we demonstrate that respiratory viral infection in the AD mouse model leads to elevated amyloid-β plaque load in the hippocampus and also induces a chronic increase in the activation status of microglia, which was not the case for non-infected APP/PS1 animals. Since these signs of neuroinflammation might indeed affect hippocampal function, we next questioned whether neuronal networks might indeed be altered.

### Dendritic Spine Density Is Reduced After H3N2 IAV Infection in APP/PS1 Mice

**FIGURE 3:**
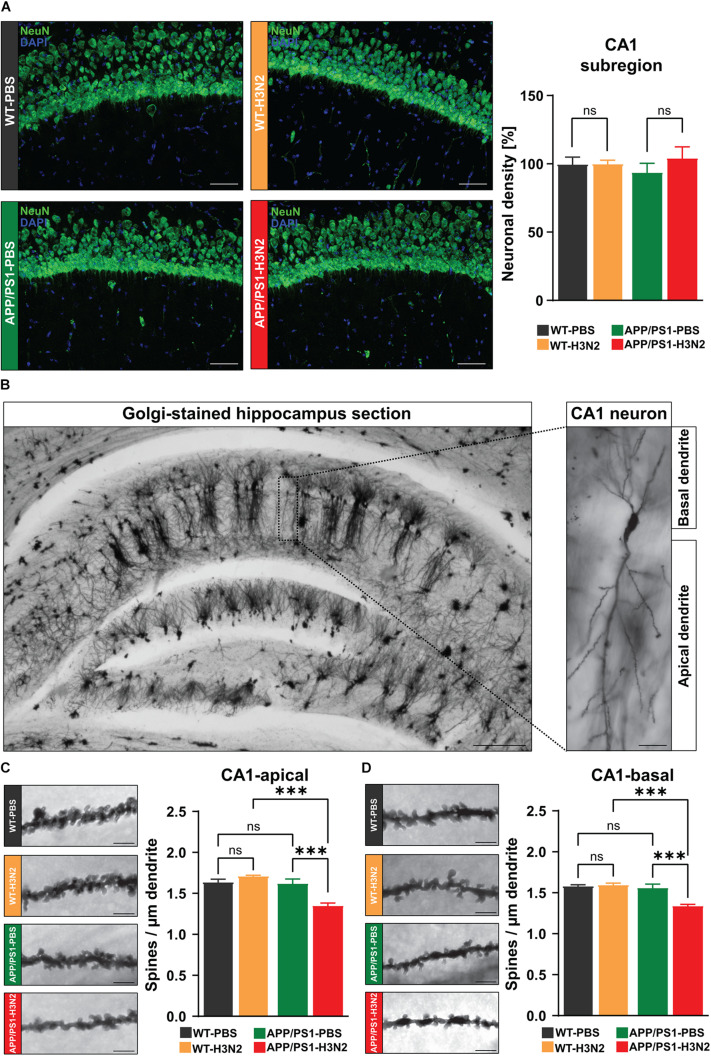
H3N2 IAV infection changes CA1 hippocampal neuron morphology in APP/PS1 mice. **(A)** Representative examples of NeuN immunostaining at 120 dpi (20X); Scale bar = 50 μm. The number of mature neurons was not altered significantly in the infected vs. non-infected groups of both genotypes [N (number of animals in each group) = 3–4, five sections were taken for each mouse and data are presented from averaging the values of the sections per animal]. **(B)** Representative images of Golgi-Cox stained hippocampus sections (2.5X); Scale bar = 200 μm and CA1 hippocampal neuron (20X); Scale bar = 20 μm. 120 days post H3N2 IAV infection, spine density of both **(C)** apical and **(D)** basal dendrites of CA1 pyramidal neurons was decreased significantly only in APP/PS1 mice. The other tested groups did not show any significant differences (*N* = 3, for each individual 10 dendrites were selected and data are presented from averaging the values of the dendrites per animal). Representative examples of dendritic spines in hippocampal CA1 neurons are shown for each group (63X); Scale bar = 2 μm. Data are presented as mean ± SEM, ***p < 0.001.

At the cellular level, AD is characterized by the degeneration of multiple neuronal populations in different brain regions including the hippocampus (DeTure and Dickson, 2019). On the other hand, infection with viruses, particularly neurotropic variants, may also result in acute and long-lasting neuronal loss (Garber et al., 2019). Therefore, to verify the role of purely peripheral immune stimulation caused by H3N2 IAV infection for progressive neuronal cell loss in APP/PS1 mice, anti-NeuN (mature neuronal marker) staining was performed (Figure 3A). Consistent with previous observations (Chaney et al., 2018), no significant differences in the number of NeuN positive cells were detected between non-infected WT and APP/PS1 mice (Δ 6.5%) at 6 months of age. There was also no evidence of cell loss after infection in either genotype [WT: Δ 0.2%, APP/PS1: Δ 4%, two-way ANOVA *F*_Genotype×Infection (1, 9)_ = 0.036, *p* = 0.85, Figure 3A].

As synapse loss is often described to precede neuronal cell loss, in the next step dendritic spine density was analyzed in CA1 pyramidal neurons using Golgi-Cox staining (Figure 3B). Dendritic spines are tiny protrusions carrying the majority of excitatory synapses in the neocortex and hippocampus, therefore, alterations in the density of these structures can be used as an index of network dysfunction (Moser et al., 1994). Dendritic spines were counted separately on apical and basal dendrites of CA1 pyramidal neurons in the hippocampus of control and infected mice in both genotypes (Figure 3B). Again in line with our previous report, dendritic spine density was not significantly altered 120 days post H3N2 IAV infection in WT mice (Apical: Δ 4%, *p* = 0.19, basal: Δ 1%, *p* = 0.72, Figures 3C,D; Hosseini et al., 2018). In contrast to this, 120 days post IAV infection APP/PS1 mice exhibited a significantly lower spine density in both apical and basal dendrites compared to non-infected APP/PS1 (apical: Δ 18%, *p* = 0.0008; basal: Δ 15%, *p* = 0.0006) and also IAV infected WT mice (apical: Δ 23%, *p* = 0.0001; basal: Δ 17%, *p* = 0.0002), [Two-way ANOVA *F*_Genotype×Infection (1, 8)_ = 21.78, *p* = 0.0016, Figure 3C; *F*_Genotype×Infection (1, 8)_ = 16.94, *p* = 0.0034, Figure 3D].

Therefore, our results indeed demonstrate that peripheral immune stimulation caused by H3N2 IAV infection is able to accelerate the occurrence of network deficits in APP/PS1 mice.

### Synaptic Plasticity Is Impaired Chronically Following H3N2 IAV Infection in APP/PS1 Mice

**FIGURE 4:**
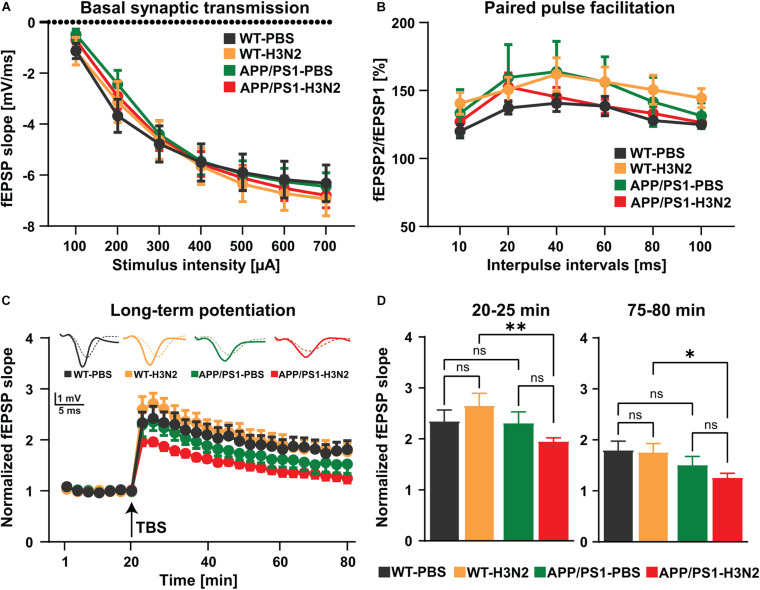
H3N2 IAV infection impairs synaptic plasticity in APP/PS1 mice. **(A)** Input-output curves of field excitatory postsynaptic potential (fEPSP) slopes in hippocampal slices obtained from all tested groups did not show any significant differences [N (number of animals in each group) = 3–4, n (number of slices in each group) = 9–13]. **(B)** The paired-pulse facilitation (PPF) of the fEPSP slopes distinct as a response to the second stimulation over the first one at different interpulse intervals (10, 20, 40, 60, 80, and 100 ms) in hippocampal slices did not exhibit any significant differences between the groups (*N* = 3–4, *n* = 9–13). **(C)** The hippocampal slices obtained from H3N2 IAV infected APP/PS1 mice showed significantly lower long-term potentiation (LTP) compared to infected WT mice. **(D)** H3N2 IAV infected APP/PS1 mice exhibited significantly reduced induction (T 20–25 min) and maintenance (T 75–80 min) of LTP compared to infected WT mice, whereas non-infected APP/PS1 mice showed slightly lower maintenance of LTP compared to non-infected WT mice which was not statistically significant (*N* = 3–4, *n* = 7–12). Data are presented as mean ± SEM of the values of the slices in each group (since it is technically impossible to always obtain the same number of slices per mouse). *p < 0.05 and **p < 0.01.

Impairments in excitatory synaptic transmission and especially synaptic plasticity are generally considered as preliminary events in the progression of AD (Volianskis et al., 2010). In order to investigate whether H3N2 IAV infection would chronically impair processes of plasticity in hippocampal networks long-term potentiation was induced by theta-burst stimulation in the Schaffer collateral pathway and recordings were performed in the stratum radiatum of the CA1 sub-region (Figure 4).

First, to assess alterations in basal synaptic transmission the dependence of the field excitatory postsynaptic potential (fEPSP) slope on stimulation intensity was analyzed in input/output curves (Figure 4A). No differences were found between experimental groups [Two-way RM ANOVA *F*_(3, 40)_ = 0.11, *p* = 0.95, Figure 4A]. This result indicates that baseline synaptic transmission was not chronically affected by viral infection even in the AD mouse model. In a second step, potential chronic impacts of H3N2 IAV infection on short-term synaptic plasticity at the Schaffer collateral-CA1 synapse were investigated using paired-pulse facilitation (PPF) (Figure 4B). PPF is a presynaptic form of short-term plasticity in which the synaptic response to the second of a pair of closely spaced stimuli is increased due to residual Ca^2+^ in the presynaptic nerve terminal from the first stimulus adding to the influx of Ca^2+^ from the second stimulus (Patterson et al., 1996). Here as well, no significant alterations were observed in all hippocampal slices obtained from the different experimental groups [Two-way RM ANOVA *F*_(3, 36)_ = 0.88, *p* = 0.45, Figure 4B]. Taken together these results revealed that basal synaptic transmission, as well as short-term synaptic plasticity in the hippocampal CA1 sub-region, were not affected in the AD mouse model at 6 months of age, and furthermore that also IAV infection did not lead to long-term alterations in WT or APP/PS1 mice (Figures 4A,B).

As a next step, long-term synaptic plasticity, the capability of synapses to change their strength, which is considered a cellular correlate of the ability to form new memories (Korte and Schmitz, 2016) was examined (Figures 4C,D). Long-term potentiation (LTP) at the Schaffer collateral CA3 to CA1 pathway was induced by theta-burst stimulation (TBS) after 20 min of stable baseline recording (Figure 4C). Comparable to what we showed previously 120 days post H3N2 IAV infection, both induction (5 min after TBS) and maintenance phase of LTP (last 5 min of the recording) were not affected in WT mice (Figures 4C,D; Hosseini et al., 2018). Moreover, 6 months old non-infected APP/PS1 mice only exhibited a slightly lower LTP maintenance compared to non-infected WT mice, however, it was not statistically significant (Figures 4C,D). In contrast, peripheral immune stimulation induced by H3N2 IAV infection resulted in chronically impaired induction (*p* = 0.009) and maintenance (*p* = 0.019) LTP in 6 months old APP/PS1 mice in comparison with WT animals [Two-way RM ANOVA *F*_(3, 33)_ = 3.60, *p* = 0.023, Figure 4C; first 5 min: *F*_Genotype(1, 33)_ = 4.30, *p* = 0.045 and last 5 min: *F*_Genotype (1, 33)_ = 7.66, *p* = 0.009, Figure 4D]. These findings further underline the pathological alterations already observed on the level of spine density thereby strengthening the point that H3N2 IAV infection may indeed accelerate the synaptic function deficits in the APP/PS1 mice.

### H3N2 IAV Infection Accelerates Cognitive Function Impairments in APP/PS1 Mice

**FIGURE 5:**
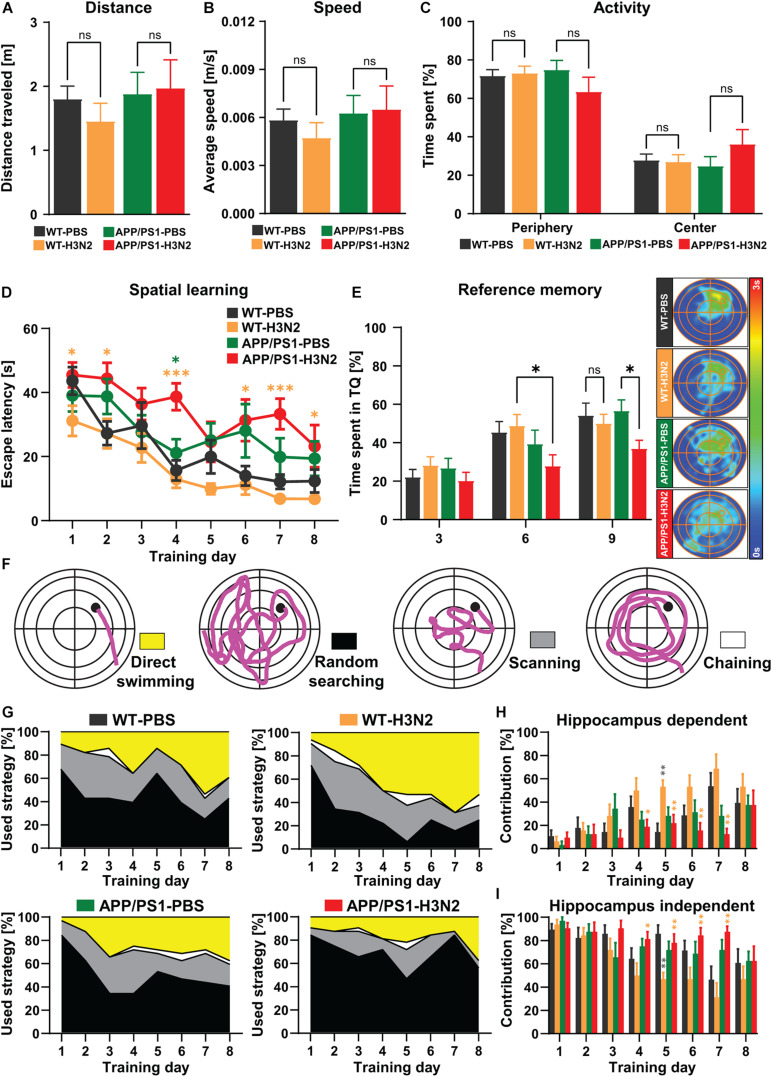
H3N2 IAV infection accelerates cognitive decline in APP/PS1 mice. **(A)** Total distance traveled, **(B)** average speed and **(C)** activity percentage of mice in the periphery and center parts of the open field arena did not indicate any significant differences between groups. **(D)** During 8 days of acquisition training, the escape latency reduced significantly in all groups, indicating spatial learning, however, the escape latency was significantly increased in H3N2 IAV infected APP/PS1 mice compared to other groups. Although 6 months old APP/PS1 mice exhibited slightly higher escape latency compared to WT mice, this was not significant. **(E)** The time spent percentage in the target quadrant (TQ, northeast) by WT and non-infected APP/PS1 did not show any significant differences, though H3N2 IAV infected APP/PS1 mice showed a significantly diminished target quadrant preference on days 6 and 9 compared to other groups. This was shown by the occupancy plots as well. **(F)** Different searching strategies including hippocampus-dependent (direct swimming) and hippocampus-independent (random searching, scanning and chaining) were shown and color-coded. **(G)** The relative contribution of the respective strategy is presented for each day of the Morris water maze test. APP/PS1 mice, at 120 days following H3N2 IAV infection utilized significantly **(H)** less hippocampal-dependent and **(I)** more hippocampal-independent searching strategies compared to infected WT animals (*N* = 7–8 in each group). Data are presented as mean ± SEM, *p < 0.05 and **p < 0.01 (orange asterisks compared to WT-H3N2, gray asterisks compared to WT-PBS, green asterisks compared to APP/PS1-PBS).

**FIGURE 6:**
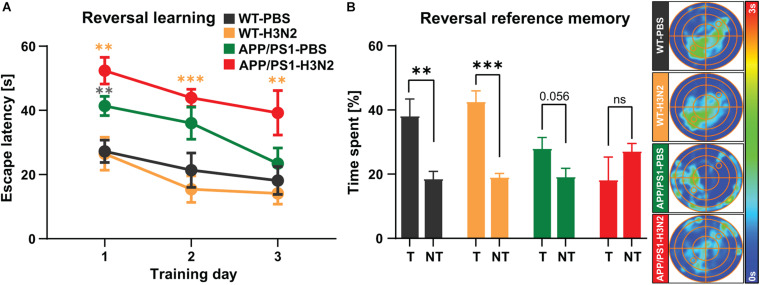
Six months old non-infected and H3N2 IAV infected APP/PS1 mice exhibit reversal learning deficits. **(A)** During 3 days of reversal learning, non-infected and H3N2 IAV infected APP/PS1 mice showed significantly higher escape latency in comparison with WT. **(B)** In the probe trial, only non-infected and infected WT mice spent significantly more time in the new target quadrant (T) compared to the average time spent in non-target quadrants (NT). Both non-infected and infected APP/PS1 mice did not show any significant preference for the new target quadrant as it was shown in the occupancy plots as well (*N* = 7–8 in each group). Data are presented as mean ± SEM, **p < 0.01 and ***p < 0.001 (orange asterisks compared to WT-H3N2, gray asterisks compared to WT-PBS).

Complex cognitive abilities such as learning and memory depend on the integrated function of neuronal systems. Therefore, the observed deficits in neuronal connectivity and synaptic plasticity may indeed result in cognitive impairments (Korte and Schmitz, 2016). Progressive memory impairment is one of the core hallmarks of AD pathogenesis which is detectable already in the early disease course (Volianskis et al., 2010; Marchetti and Marie, 2011). Moreover, it was shown that systemic and central viral infection may induce learning and memory deficits not only during the acute phase but also chronically after the virus in cleared (Jurgens et al., 2012; Hosseini et al., 2018; Garber et al., 2019). Therefore, to identify potential long-term impacts of H3N2 IAV infection on the progression of cognitive impairments in APP/PS1 mice, learning and memory formation were assessed using the Morris water maze (MWM) paradigm (Figures 5, 6).

General locomotion, exploration and anxiety-related behavior of mice were assessed prior to the MWM training in the open-field test (Figures 5A–C) to exclude that phenotypes detected in the MWM would be purely attributable to deficits in locomotor or anxiety-related behavior, especially following infection. As was shown also previously, 120 days post H3N2 IAV infection, no significant changes in the total distance traveled (Figure 5A), average speed (Figure 5B) and general activity in the periphery and center zones of the open-field arena (Figure 5C) were detected in WT mice (Hosseini et al., 2018). Likewise, non-infected and infected APP/PS1 mice as well did not show any obvious locomotion impairment or anxiety-related behavior. Overall total distance traveled [Two-way ANOVA: *F*_Genotype(1, 27)_ = 0.76, *p* = 0.39; *F*_Infection(1, 27)_ = 0.14, *p* = 0.70; *F*_Genotype×Infection(1, 27)_ = 0.42, *p* = 0.52, Figure 5A], average speed [Two-way ANOVA: *F*_Genotype (1, 27)_ = 0.92, *p* = 0.34; *F*_Infection (1, 27)_ = 0.15, *p* = 0.70; *F*_Genotype×Infection (1, 27)_ = 0.37, *p* = 0.54, Figure 5B] and time spent in the periphery vs. center parts of the arena [Two-way ANOVA *F*_(3, 54)_ = 5.06, *p* > 0.99, Figure 5C] were comparable between all tested groups.

During 8 days of acquisition in the MWM, the escape latency (time to reach the hidden platform located in NE quadrant) progressively decreased in a significant manner in all control and H3N2 IAV infected groups at 120 dpi (Figure 5D) indicating hippocampus-dependent spatial learning [One-way RM ANOVA *F*_wT–PBS(7, 42)_ = 12.62, *p* < 0.0001; *F*_wT–H3N2(7, 49)_ = 10.17, *p* < 0.0001; *F*_aPP/PS1–PBS(7, 49)_ = 4.32, *p* = 0.0009; *F*_aPP/PS1–H3N2 (7, 49)_ = 3.47, *p* = 0.0042]. 120 days post H3N2 IAV infection, the escape latency was not altered significantly in infected WT mice compared to the WT control group. The escape latency was slightly higher in 6 months old non-infected APP/PS1 mice compared to WT indicating a mild impairment which was, however, not significant. Notably, APP/PS1 animals trained 120 days post H3N2 IAV infection showed a significant increase in the escape latency especially on day 4 compared to the non-infected APP/PS1 (*p* = 0.011) and infected WT mice (*p* = 0.0002), [Two-way RM ANOVA *F*_(3, 27)_ = 5.70, *p* = 0.003, Figure 5D]. To further analyze memory retrieval, reference memory tests (without a platform) on day 3 and 6 prior to the training and on day 9, 24 h after the last training, were carried out (Figure 5E). As was the case for the learning, the results of the probe trials indicated that the time spent in the target quadrant by H3N2 IAV infected APP/PS1 mice was significantly diminished on day 6 and 9 compared to infected WT (*p* = 0.02) and non-infected APP/PS1 mice (*p* = 0.014), respectively [Two-way RM ANOVA, *F*_(3, 27)_ = 3.25, *p* = 0.036, Figure 5E], clearly showing an impairment in memory retrieval. The performance in all other groups was comparable (Figure 5E).

To further investigate the quality of spatial learning during the acquisition training, the searching strategies were evaluated. A detailed analysis of the swimming path in the middle of the pool, the outer region, an annulus doughnut-shaped zone and the Wishaw’s corridor allows to differentiate the searching strategies used by the mice into hippocampus-dependent (direct swimming) and hippocampus-independent strategies (random searching, scanning and chaining) (Figure 5F). Progressively over time, healthy animals switch from egocentric (hippocampus-independent) to allocentric (hippocampus-dependent) strategies to locate the hidden platform (Garthe et al., 2009; Garthe and Kempermann, 2013). As expected the relative contribution of hippocampus-dependent strategies increased over the 8 days of training in all tested groups (Figure 5G). However, this progression was diminished in non-infected as well as in infected APP/PS1 mice compared to WT animals (Figure 5G). Also, further quantification showed that H3N2 IAV infected APP/PS1 mice utilized less hippocampus-dependent and more hippocampus-independent searching strategies, especially compared to infected WT mice which were more pronounced between days 4 and 7 of acquisition [Two-way RM ANOVA *F*_(3, 27)_ = 3.38, *p* = 0.032, Figures 5H,I]. At 120 dpi, WT infected mice and WT non-infected mice used comparable searching strategies, with the exception of day 5, when infected WT mice used more hippocampus-dependent and less -independent strategies (*p* = 0.0014) (Figures 5H,I).

After the initial spatial acquisition, a reversal training was performed (Figure 6). In this test the hidden platform was moved to the opposite quadrant (SW) and the mice were trained for another 3 days. Solving this test is more difficult and needs behavioral flexibility which requires animals to update the new platform location in their spatial map (Vorhees and Williams, 2006). The findings of the reversal learning test showed that the escape latency was not altered in the WT animals at 120 days post infection compared to non-infected WT mice. However, non-infected as well as H3N2 IAV infected APP/PS1 mice exhibited a higher escape latency compared to non-infected and infected WT mice, respectively [Two-way RM ANOVA, *F*_(3, 27)_ = 11.09, *p* < 0.001, Figure 6A]. Twenty four hours after the last reversal training, another probe trial was performed (Figure 6B). As was the case for reversal learning, only WT non-infected (Unpaired *t*-test: *t* = 3.45, df = 12, *p* = 0.004) and WT infected mice (Unpaired *t*-test: *t* = 6.78, df = 14, *p* < 0.001) spent significantly more time in the new target quadrant (T) compared to mean time spent in the non-target quadrants (NT). Non-infected APP/PS1 mice (Unpaired *t*-test: *t* = 2.08, df = 14, *p* = 0.056) as well as H3N2 IAV infected APP/PS1 animals (Unpaired *t*-test: *t* = 1.20, df = 14, *p* = 0.24) did not show any significant preference for the new target quadrant (Figure 6B).

Taken together, we can show that at 6 months of age learning impairments can be observed in the AD mouse model which became more pronounced 120 days after H3N2 IAV infection.

## Discussion

Alzheimer’s disease (AD), the most common cause of dementia, is an inherited or sporadic age-related disorder characterized by progressive cognitive decline. Less than 1% of people under the age of 65 carry the genetic mutations for AD, therefore, aging can be considered the leading risk factor for AD. However, the exact etiology of AD still remains mostly elusive (Guerreiro and Bras, 2015). One of the important pathological hallmarks of AD is an extracellular accumulation of amyloid-β which is closely associated with an inflammatory response and subsequent neurodegeneration that might indeed be responsible for the loss of memory in AD (Heneka et al., 2015). Interestingly, recent investigations suggest that amyloid-β production and deposition are not unique for AD and may also be triggered in the CNS through other pathological conditions such as infections. For instance, HHV-1 infection forces H4 neuroglioma cells to produce amyloid-β_42_ peptide preventing secondary viral replication (Bourgade et al., 2016). Furthermore, amyloid-β_42_ administration has been shown to be able to inhibit H1N1 and H3N2 influenza A virus (IAV) replication *in vitro*, indicating antiviral properties of amyloid-β that modulate viral interaction with phagocytes (White et al., 2014). Hence, an inappropriate immune response in the brain may be engaged in triggering or further exacerbating neuropathological processes associated with AD. Indeed, increasing evidence points toward a critical role of neuroinflammation in the etiopathogenesis of AD. Accordingly, infectious agents in the CNS itself or also merely in the periphery can stimulate the activation of microglia and astrocytes, thereby inducing neuroinflammation, which in turn leads to the neuropathological changes observed in AD (Sochocka et al., 2017).

Infection with respiratory viruses including IAV represents a major threat to human and animal health today and possesses the potential for worldwide severe pandemics (Bohmwald et al., 2018). Although the primary effect of IAV occurs in the respiratory system, some previous clinical and basic studies have addressed neurological sequelae induced by IAV (Ekstrand, 2012). Given the increased risk of respiratory viral infections due to spreading in an increasingly globally connected human population, it is of high relevance to reveal and understand potentially chronic neurological manifestations following IAV infection.

Our recent findings provide evidence that microglial activation caused by non-neurotropic H3N2 and neurotropic H7N7 IAV infection induces long-lasting impairments in hippocampal function and cognition in young adult animals at 30 dpi, which, however, recovered 3 months later (Hosseini et al., 2018). These findings were also reproduced in the current study. We therefore asked the question if long-term neuroinflammation induced by a peripheral IAV infection might indeed be able to or enhance the development of neurodegenerative diseases. In this respect, it was shown that H1N1 and H5N1 IAV infections can initiate neuronal pathology and protein aggregation similar to that observed in proteinopathies including Parkinson’s and Alzheimer’s diseases (Jang et al., 2009; Marreiros et al., 2020).

In the APP/PS1 transgenic AD mouse model used in this study pathology starts around 6 months of age when the Aβ burden increases (Trinchese et al., 2004). Therefore, 2 months old APP/PS1 mice well before the onset of symptoms were infected with the H3N2 IAV subtype and analyzed 4 months later (at 120 dpi). Already young mice, in the absence of AD pathology, were more susceptible to IAV infection because they survived less, lost more bodyweight and recovered more slowly than WT mice. It was shown previously that patients with dementia more frequently suffer from respiratory infections, a fact that also now can be observed in the recent COVID-19 pandemic (Alonso-Lana et al., 2020). Yet, the exact mechanisms remain unknown. It is noteworthy that a metabolomics study performed in the spleen and thymus of 6 months old APP/PS1 mice revealed significant differences in numerous metabolites compared to WT mice. Several biochemical pathways involved in the homeostasis of amino acids and oxidative stress were altered, indicating that 6 months old APP/PS1 mice have significant impairments in the peripheral immune system (González-Domínguez et al., 2015). Interestingly, Yu et al. (2017) investigated metabolic changes in the urine of APP/PS1 transgenic mice prior to cognitive decline at 2 months of age in which significant alterations of several metabolites compared to WT mice were detected. These findings suggest that early metabolic changes occur in APP/PS1 mice even prior to the onset of common disease symptoms (Yu et al., 2017). Therefore, even in the preclinical stage of AD pathology, individuals may already have a compromised immune response that does not allow them to cope successfully with viral infections.

In contrast to the sparse knowledge about the role of the peripheral immune response for AD, immunological mechanisms associated with AD pathology directly in the CNS are better described. Amyloid-β protein aggregations bind to pattern recognition receptors on microglial cells and lead to microglial proliferation and activation as well as subsequent excessive release of inflammatory mediators such as TNF-α, IL-1β, and IL-6 which contribute to disease progression and severity (Heneka et al., 2015). In line with this, our results showed that microglial density was already increased in the hippocampus of non-infected APP/PS1 mice, albeit with no signs of increased activation. Infection with H3N2 IAV, however, enhanced this phenotype on a long time scale also leading to a higher activation status of microglia in 6 months old APP/PS1 mice. In this respect, it is important to mention that inflammatory cytokines generated during peripheral inflammation can activate a secondary, mirror inflammatory response by activation of microglia in the brain that is characterized by the production of proinflammatory cytokines (Riazi et al., 2015). Therefore, acceleration of microglial activation in the hippocampus of APP/PS1 mice might at least in part be attributable to the initial peripheral immune response which might be transmitted to the CNS via a leaky blood-brain-barrier (BBB) induced by H3N2 IAV infection during the acute phase of the infection (Hosseini et al., 2018).

Although microglial activation is critical for the host defense against pathogenesis, excessive activation can have devastating impacts on CNS integrity. In AD, activated microglia with loss of key homeostatic functions can be detrimental for neuronal function and survival. In 6 months old APP/PS1 mice an increase in microglial activation was detectable following H3N2 IAV infection, however, this was not (or not yet) associated with significant neuronal loss. Indeed, neuronal loss is not considered as an early event in AD pathogenesis which has been confirmed in several AD mouse models (Wirths and Bayer, 2010). Among the initial steps of AD progression, an age-dependent loss in the number of dendritic spines is described which often precedes cell loss. Interestingly whereas non-infected APP/PS1 mice at 6 months of age did not show signs of synapse loss or impairments in synaptic plasticity yet a history of IAV infection indeed seemed to have triggered a faster progression of disease symptoms in infected APP/PS1 animals. Previous studies using different AD transgenic mouse models indicate that especially the onset of the disease phenotype is variable with some being able to detect already mild alterations in synapse number or synaptic plasticity at this age whereas others did not yet find differences (Gengler et al., 2010; Perez-Cruz et al., 2011; Viana da Silva et al., 2016). This further emphasizes that previous infection with H3N2 IAV increased disease progression thereby resulting in clearly detectable impairments compared to non-infected APP/PS1 mice.

Previously, it was shown that microglia become acutely activated upon a peripheral immune challenge in APP/PS1 mice. Two-photon *in vivo* laser scanning microscopy showed a comparable level of activation of microglia 2 days upon an LPS challenge in both 5 months and 15 months old APP/PS1 mice. Importantly, the activation status of microglia in transgenic mice without LPS was only altered in 15 months old mice but not at 5 months of age (Tejera et al., 2019). Peripheral immune stimulation in APP/PS1 mice might therefore chronically activate microglia in an age-dependent manner maybe even lasting for months in the AD mouse model, and this might very well precede cognitive manifestations. On the other hand, systemic inflammation reduces microglial clearance of amyloid-β in APP/PS1 mice (Tejera et al., 2019). Thus reduced Aβ clearance capacity during the initial disease course may underlie further microglial activation triggered by the release of pro−inflammatory cytokines and subsequent dendritic spine loss and impaired synaptic plasticity which were seen only in H3N2 IAV infected APP/PS1 mice at 6 months of age.

Microglial activation and accumulation of amyloid-β plaques can be associated with an increase in the severity of cognitive impairments in AD (Heneka et al., 2015). Our assessment of cognitive function showed already mild deficits in non-infected APP/PS1 mice at 6 months of age, especially during reversal learning. In line with the data describing a reduction in spine density and impairment in synaptic plasticity, this was more pronounced in H3N2 IAV infected APP/PS1 mice. Previous findings suggested that the progression of amyloidosis and cognitive decline must be accompanied by progressive loss of synapses and impairment in plasticity. Interestingly, APP/PS1 mice at 6 months of age showed albeit mild episodic spatial memory deficits without obvious signs of cellular decline as synapse loss or LTP impairments. Therefore, accumulation of amyloid-β and episodic memory deficits, both of which increased with age in APP/PS1 mice, are not always directly accompanied by alterations in synaptic transmission or synapse number as has also been shown previously (Volianskis et al., 2010). Since LTP impairments can indeed be detected in CA3 neurons in 6 months old APP/PS1 mice (Viana da Silva et al., 2016), our results may suggest that the neuronal representation of behavioral flexibility to decide between competing memories in the reversal learning may reside more in the CA3 hippocampal sub-region.

Taken together, our findings strongly point toward the fact that purely peripheral immune stimulation induced by H3N2 IAV may commence inflammatory processes in the brain via microglial activation and therefore accelerate the progression of Alzheimer’s disease.

Currently, there is insufficient evidence to know whether vaccination would be an efficient strategy to prevent severe consequences of an immune challenge in AD patients, but for many reasons this might be an avenue to take. With regard to the complex crosstalk between the nervous system and the immune system, further research is needed to shed light on the role of infections for AD pathogenesis.

## Data Availability Statement

The original contributions presented in the study are included in the article/Supplementary Material, further inquiries can be directed to the corresponding author/s.

## Ethics Statement

The animal study was reviewed and approved by the local committees at the Helmholtz Centre for Infection Research and TU Braunschweig and the authorities (LAVES, Oldenburg, Germany; permit number: 18/2968) according to the national guidelines of the animal welfare law in Germany.

## Author Contributions

SH, KM-P, and MK designed research, analyzed data, and wrote the manuscript. SH performed research. KS contributed new reagents and analytic tools. All authors contributed to the article and approved the submitted version.

## Conflict of Interest

The authors declare that the research was conducted in the absence of any commercial or financial relationships that could be construed as a potential conflict of interest.

## References

[B1] Allnutt M. A. Johnson K. Bennett D. A. Connor S. M. Troncoso J. C. Pletnikova O. (2020). Human herpesvirus 6 detection in Alzheimer’s disease cases and controls across multiple cohorts. *Neuron* 105 1027–1035.e2. 10.1016/j.neuron.2019.12.031 31983538 PMC7182308

[B2] Alonso-Lana S. Marquié M. Ruiz A. Boada M. (2020). Cognitive and neuropsychiatric manifestations of COVID-19 and effects on elderly individuals with dementia. *Front. Aging Neurosci.* 12:588872. 10.3389/fnagi.2020.588872 33192483 PMC7649130

[B3] Bohmwald K. Gálvez N. M. S. Ríos M. Kalergis A. M. (2018). Neurologic alterations due to respiratory virus infections. *Front. Cell Neurosci.* 12:386. 10.3389/fncel.2018.00386 30416428 PMC6212673

[B4] Bourgade K. Le Page A. Bocti C. Witkowski J. M. Dupuis G. Frost E. H. (2016). Protective effect of amyloid-β peptides against herpes simplex virus-1 infection in a neuronal cell culture model. *J. Alzheimers. Dis.* 50 1227–1241. 10.3233/jad-150652 26836158

[B5] Bowery N. G. Bagetta G. Nisticó G. Britton P. Whitton P. (1992). Intrahippocampal tetanus toxin produces generalized convulsions and neurodegeneration in rats: antagonism by NMDA receptor blockers. *Epilepsy Res. Suppl.* 9 249–256. 1363043

[B6] Chaney A. Bauer M. Bochicchio D. Smigova A. Kassiou M. Davies K. E. (2018). Longitudinal investigation of neuroinflammation and metabolite profiles in the APP(swe) ×PS1(Δe9) transgenic mouse model of Alzheimer’s disease. *J. Neurochem.* 144 318–335. 10.1111/jnc.14251 29124761 PMC5846890

[B7] De Chiara G. Marcocci M. E. Sgarbanti R. Civitelli L. Ripoli C. Piacentini R. (2012). Infectious agents and neurodegeneration. *Mol. Neurobiol.* 46 614–638. 10.1007/s12035-012-8320-8327 22899188 PMC3496540

[B8] DeTure M. A. Dickson D. W. (2019). The neuropathological diagnosis of Alzheimer’s disease. *Mol. Neurodegeneration* 14:32. 10.1186/s13024-019-0333-335 PMC6679484 31375134

[B9] Ekstrand J. J. (2012). Neurologic complications of influenza. *Semin. Pediatr Neurol.* 19 96–100. 10.1016/j.spen.2012.02.004 22889537

[B10] Garber C. Soung A. Vollmer L. L. Kanmogne M. Last A. Brown J. (2019). T cells promote microglia-mediated synaptic elimination and cognitive dysfunction during recovery from neuropathogenic flaviviruses. *Nat. Neurosci.* 22 1276–1288. 10.1038/s41593-019-0427-y 31235930 PMC6822175

[B11] Garthe A. Behr J. Kempermann G. (2009). Adult-generated hippocampal neurons allow the flexible use of spatially precise learning strategies. *PLoS One* 4:e5464. 10.1371/journal.pone.0005464 19421325 PMC2674212

[B12] Garthe A. Kempermann G. (2013). An old test for new neurons: refining the Morris water maze to study the functional relevance of adult hippocampal neurogenesis. *Front. Neurosci.* 7:63. 10.3389/fnins.2013.00063 23653589 PMC3642504

[B13] Gengler S. Hamilton A. Hölscher C. (2010). Synaptic plasticity in the hippocampus of a APP/PS1 mouse model of Alzheimer’s disease is impaired in old but not young mice. *PLoS One* 5:e9764. 10.1371/journal.pone.0009764 20339537 PMC2842299

[B14] González-Domínguez R. García-Barrera T. Vitorica J. Gómez-Ariza J. L. (2015). Metabolomics reveals significant impairments in the immune system of the APP/PS1 transgenic mice of Alzheimer’s disease. *Electrophoresis* 36 577–587. 10.1002/elps.201400450 25393935

[B15] Guerreiro R. Bras J. (2015). The age factor in Alzheimer’s disease. *Genome Med.* 7:106. 10.1186/s13073-015-0232-235 PMC4617238 26482651

[B16] Haller O. Arnheiter H. Lindenmann J. (1979). Natural, genetically determined resistance toward influenza virus in hemopoietic mouse chimeras. role mononuclear phagocytes. *J. Exp. Med.* 150 117–126. 10.1084/jem.150.1.117 36443 PMC2185613

[B17] Heneka M. T. Golenbock D. T. Latz E. (2015). Innate immunity in Alzheimer’s disease. *Nat. Immunol.* 16 229–236. 10.1038/ni.3102 25689443

[B18] Hosseini S. Michaelsen-Preusse K. Grigoryan G. Chhatbar C. Kalinke U. Korte M. (2020). Type I interferon receptor signaling in astrocytes regulates hippocampal synaptic plasticity and cognitive function of the healthy CNS. *Cell Reports* 31:107666. 10.1016/j.celrep.2020.107666 32433975

[B19] Hosseini S. Wilk E. Michaelsen-Preusse K. Gerhauser I. Baumgärtner W. Geffers R. (2018). Long-Term neuroinflammation induced by influenza a virus infection and the impact on hippocampal neuron morphology and function. *J. Neurosci.* 38 3060–3080. 10.1523/jneurosci.1740-17.2018 29487124 PMC6596076

[B20] Jang H. Boltz D. Sturm-Ramirez K. Shepherd K. R. Jiao Y. Webster R. (2009). Highly pathogenic H5N1 influenza virus can enter the central nervous system and induce neuroinflammation and neurodegeneration. *Proc. Natl. Acad. Sci. U S A.* 106 14063–14068. 10.1073/pnas.0900096106 19667183 PMC2729020

[B21] Jiruska P. Shtaya A. B. Y. Bodansky D. M. S. Chang W.-C. Gray W. P. Jefferys J. G. R. (2013). Dentate gyrus progenitor cell proliferation after the onset of spontaneous seizures in the tetanus toxin model of temporal lobe epilepsy. *Neurobiol. Dis.* 54 492–498. 10.1016/j.nbd.2013.02.001 23439313 PMC3635088

[B22] Jurgens H. A. Amancherla K. Johnson R. W. (2012). Influenza infection induces neuroinflammation, alters hippocampal neuron morphology, and impairs cognition in adult mice. *J. Neurosci.* 32 3958–3968. 10.1523/jneurosci.6389-11.2012 22442063 PMC3353809

[B23] Kinney J. W. Bemiller S. M. Murtishaw A. S. Leisgang A. M. Salazar A. M. Lamb B. T. (2018). Inflammation as a central mechanism in Alzheimer’s disease. *Alzheimers Dement (N Y)* 4 575–590. 10.1016/j.trci.2018.06.014 30406177 PMC6214864

[B24] Kollmus H. Pilzner C. Leist S. R. Heise M. Geffers R. Schughart K. (2018). Of mice and men: the host response to influenza virus infection. *Mamm. Genome* 29 446–470. 10.1007/s00335-018-9750-y 29947965 PMC6132725

[B25] Korte M. Schmitz D. (2016). Cellular and system biology of memory: timing. *Mol. Beyond. Physiol. Rev.* 96 647–693. 10.1152/physrev.00010.2015 26960344

[B26] Leist S. R. Pilzner C. van den Brand J. M. Dengler L. Geffers R. Kuiken T. (2016). Influenza H3N2 infection of the collaborative cross founder strains reveals highly divergent host responses and identifies a unique phenotype in CAST/EiJ mice. *BMC Genomics* 17:143. 10.1186/s12864-016-2483-y 26921172 PMC4769537

[B27] Liu P. P. Xie Y. Meng X. Y. Kang J. S. (2019). History and progress of hypotheses and clinical trials for Alzheimer’s disease. *Signal Transduct Target Ther.* 4:29. 10.1038/s41392-019-0063-68 PMC6799833 31637009

[B28] Mandrekar-Colucci S. Landreth G. E. (2010). Microglia and inflammation in Alzheimer’s disease. *CNS Neurol. Disord. Drug Targets* 9 156–167. 10.2174/187152710791012071 20205644 PMC3653290

[B29] Marchetti C. Marie H. (2011). Hippocampal synaptic plasticity in Alzheimer’s disease: what have we learned so far from transgenic models? *Rev. Neurosci.* 22 373–402. 10.1515/rns.2011.035 21732714

[B30] Marreiros R. Müller-Schiffmann A. Trossbach S. V. Prikulis I. Hänsch S. Weidtkamp-Peters S. (2020). Disruption of cellular proteostasis by H1N1 influenza a virus causes α-synuclein aggregation. *Proc. Natl. Acad. Sci. U S A.* 117 6741–6751. 10.1073/pnas.1906466117 32152117 PMC7104400

[B31] Masurkar A. V. (2018). Towards a circuit-level understanding of hippocampal CA1 dysfunction in Alzheimer’s disease across anatomical axes. *J. Alzheimers Dis. Parkinsonism* 8:412. PMC6005196 29928558

[B32] Morris R. (1984). Developments of a water-maze procedure for studying spatial learning in the rat. *J. Neurosci. Methods* 11 47–60. 6471907 10.1016/0165-0270(84)90007-4

[B33] Moser M. B. Trommald M. Andersen P. (1994). An increase in dendritic spine density on hippocampal CA1 pyramidal cells following spatial learning in adult rats suggests the formation of new synapses. *Proc. Natl. Acad. Sci. U S A.* 91 12673–12675. 10.1073/pnas.91.26.12673 7809099 PMC45501

[B34] Papageorgiou I. E. Fetani A. F. Lewen A. Heinemann U. Kann O. (2015). Widespread activation of microglial cells in the hippocampus of chronic epileptic rats correlates only partially with neurodegeneration. *Brain Struct. Funct.* 220 2423–2439. 10.1007/s00429-014-0802-800 24878824

[B35] Patterson S. L. Abel T. Deuel T. A. Martin K. C. Rose J. C. Kandel E. R. (1996). Recombinant BDNF rescues deficits in basal synaptic transmission and hippocampal LTP in BDNF knockout mice. *Neuron* 16 1137–1145. 10.1016/s0896-6273(00)80140-80143 8663990

[B36] Perez-Cruz C. Nolte M. W. van Gaalen M. M. Rustay N. R. Termont A. Tanghe A. (2011). Reduced spine density in specific regions of CA1 pyramidal neurons in two transgenic mouse models of Alzheimer’s disease. *J. Neurosci.* 31 3926–3934. 10.1523/jneurosci.6142-10.2011 21389247 PMC6622797

[B37] Perkins D. (2005). Virus signaling and apoptosis in the central nervous system infection. *Front. Biosci.* 10:2804–2819. 10.2741/1737 15970535

[B38] Rana A. Musto A. E. (2018). The role of inflammation in the development of epilepsy. *J. Neuroinflamm.* 15:144. 10.1186/s12974-018-1192-1197 PMC5952578 29764485

[B39] Riazi K. Galic M. A. Kentner A. C. Reid A. Y. Sharkey K. A. Pittman Q. J. (2015). Microglia-dependent alteration of glutamatergic synaptic transmission and plasticity in the hippocampus during peripheral inflammation. *J. Neurosci.* 35 4942–4952. 25810524 10.1523/JNEUROSCI.4485-14.2015 PMC6705378

[B40] Sadasivan S. Zanin M. O’Brien K. Schultz-Cherry S. Smeyne R. J. (2015). Induction of microglia activation after infection with the non-neurotropic A/CA/04/2009 H1N1 influenza virus. *PLoS One* 10:e0124047. 10.1371/journal.pone.0124047 25861024 PMC4393251

[B41] Sochocka M. Zwolińska K. Leszek J. (2017). The infectious etiology of Alzheimer’s Disease. *Curr. Neuropharmacol.* 15 996–1009. 10.2174/1570159x15666170313122937 28294067 PMC5652018

[B42] Tejera D. Mercan D. Sanchez-Caro J. M. Hanan M. Greenberg D. Soreq H. (2019). Systemic inflammation impairs microglial Aβ clearance through NLRP3 inflammasome. *EMBO J.* 38:e101064. 10.15252/embj.2018101064 31359456 PMC6717897

[B43] Trinchese F. Liu S. Battaglia F. Walter S. Mathews P. M. Arancio O. (2004). Progressive age-related development of Alzheimer-like pathology in APP/PS1 mice. *Ann. Neurol.* 55 801–814. 10.1002/ana.20101 15174014

[B44] van Heeckeren A. M. Tscheikuna J. Walenga R. W. Konstan M. W. Davis P. B. Erokwu B. (2000). Effect of *Pseudomonas* infection on weight loss, lung mechanics, and cytokines in mice. *Am. J. Respir. Crit. Care Med.* 161 271–279. 10.1164/ajrccm.161.1.9903019 10619831

[B45] Vasek M. J. Garber C. Dorsey D. Durrant D. M. Bollman B. Soung A. (2016). A complement-microglial axis drives synapse loss during virus-induced memory impairment. *Nature* 534 538–543. 10.1038/nature18283 27337340 PMC5452615

[B46] Viana da Silva S. Haberl M. G. Zhang P. Bethge P. Lemos C. (2016). Early synaptic deficits in the APP/PS1 mouse model of Alzheimer’s disease involve neuronal adenosine A2A receptors. *Nat. Commun.* 7:11915. 10.1038/ncomms11915 27312972 PMC4915032

[B47] Volianskis A. Køstner R. Mølgaard M. Hass S. Jensen M. S. (2010). Episodic memory deficits are not related to altered glutamatergic synaptic transmission and plasticity in the CA1 hippocampus of the APPswe/PS1δE9-deleted transgenic mice model of ß-amyloidosis. *Neurobiol. Aging* 31 1173–1187. 10.1016/j.neurobiolaging.2008.08.005 18790549

[B48] Vorhees C. V. Williams M. T. (2006). Morris water maze: procedures for assessing spatial and related forms of learning and memory. *Nat. Protoc.* 1 848–858. 10.1038/nprot.2006.116 17406317 PMC2895266

[B49] Walsh R. N. Cummins R. A. (1976). The open-field test: a critical review. *Psychol. Bull.* 83:482. 17582919

[B50] White M. R. Kandel R. Tripathi S. Condon D. Qi L. Taubenberger J. (2014). Alzheimer’s associated β-amyloid protein inhibits influenza a virus and modulates viral interactions with phagocytes. *PLoS One* 9:e101364. 10.1371/journal.pone.0101364 24988208 PMC4079246

[B51] Wirths O. Bayer T. A. (2010). Neuron loss in transgenic mouse models of Alzheimer’s disease. *Int. J. Alzheimers Dis.* 2010:723782. 10.4061/2010/723782 20871861 PMC2943100

[B52] Wolf S. A. Boddeke H. Kettenmann H. (2017). Microglia in physiology and disease. *Annu. Rev. Physiol.* 79 619–643. 27959620 10.1146/annurev-physiol-022516-034406

[B53] Yu J. Kong L. Zhang A. Han Y. Liu Z. Sun H. (2017). High-throughput metabolomics for discovering potential metabolite biomarkers and metabolic mechanism from the APPswe/PS1dE9 transgenic model of Alzheimer’s disease. *J. Proteome Res.* 16 3219–3228. 28753016 10.1021/acs.jproteome.7b00206

